# DNA Methylation in Adipose Tissue and Metabolic Syndrome

**DOI:** 10.3390/jcm9092699

**Published:** 2020-08-21

**Authors:** Sunil K. Panchal, Lindsay Brown

**Affiliations:** 1 School of Science, Western Sydney University, Richmond, NSW 2753, Australia; S.Panchal@westernsydney.edu.au; 2 Functional Foods Research Group and School of Health and Wellbeing, University of Southern Queensland, Ipswich, QLD 4305, Australia

**Keywords:** DNA methylation, metabolic syndrome, adipose tissue, epigenetics

Epigenetics is the study of heritable phenotype changes that do not involve alterations in the DNA sequence with the processes including DNA methylation, histone modifications and RNA-associated silencing [1,2]. Castellano-Castillo and colleagues have analysed the impact of metabolic syndrome on the global DNA methylation pattern and the DNA methylation of several genes in visceral adipose tissue [3]. They concluded that DNA methylation in adipose tissue of these patients is related to the aetiology of the syndrome. The role of DNA methylation in genes for adipogenesis, inflammation and lipid metabolism provides logical explanations for these changes in the development and progression of metabolic syndrome [3].

DNA methylation, occurring predominantly at sites where a cytosine nucleotide is followed by a guanine nucleotide (CpG sites) to produce 5-methylcytosine, is a mechanism by which cells can control gene expression [4]. CpG sites, located in or near gene promoters, are hypo-methylated, while other DNA regions are hyper-methylated [5]. DNA methylation has been implicated in the development of cancers and type 2 diabetes [2,6,7,8]. Epigenetic modifications of DNA may control intrauterine growth and development, along with metabolism [2,9,10,11,12].

DNA methylation plays a vital role in gene expression through the recruitment of gene repression proteins or the suppression of the binding of transcription factors [13]. Through these mechanisms, DNA methylation can change metabolism towards a healthy state or to the development of chronic metabolic disease states such as metabolic syndrome. Castellano-Castillo and colleagues [3] found positive correlations between the PPARA (gene encoding peroxisome proliferator-activated receptor-α (PPAR-α)) DNA methylation level and the metabolic syndrome index, triglyceride concentrations and HOMA-IR (HOmeostatic Model Assessment for Insulin Resistance). PPAR-α is a nuclear receptor expressed in active metabolic tissues such as liver, heart, brown fat and skeletal muscle, where it regulates the expression of genes involved in lipid metabolism [14,15]. The presence of hepatosteatosis and elevated circulating triglycerides and cholesterol, as well as increased gonadal fat in *Ppara*-null mice, suggests its significance in lipid metabolism [16,17]. Moreover, PPAR-α agonists normalise atherogenic lipid profiles and cardiovascular risk markers associated with the metabolic syndrome and type 2 diabetes [18]. Thus, epigenetic mechanisms including DNA methylation to increase PPARA gene expression may reduce the risk of metabolic syndrome. Castellano-Castillo and colleagues [3] also identified a negative association between the DNA methylation levels of the gene encoding PPAR-γ (PPARG) and diastolic blood pressure and a positive correlation between PPARG DNA methylation levels and body mass index. PPAR-γ, highly expressed in adipose tissue, plays vital roles in the regulation of adipocyte differentiation, lipid storage and glucose metabolism [14,19].

Retinoid X receptor (RXR)-α is an important factor that forms a heterodimer with PPAR-α and PPAR-α receptors. This study found a negative correlation between adipose tissue RXRA methylation and the body mass index and waist circumference [3]. Further, DNA methylation in low-density lipoprotein receptor-related protein 1 (LRP1) and lipoprotein lipase (LPL) showed positive association with metabolic syndrome variables indicating the role of epigenetics in the regulation of lipid metabolism while stearoyl-CoA desaturase (SCD1) showed negative association [3]. SCD1 is an important metabolic enzyme catalysing the synthesis of monounsaturated fatty acids from saturated fatty acids and a key regulator of carbohydrate and lipid metabolism in adipose tissue [20]. SCD1 deletion knockout mice models have confirmed that palmitoleate and oleate, products of SCD, play differential roles in controlling lipid metabolism [20].

Adipose tissue inflammation is an important factor in the development of obesity and metabolic syndrome [21]. This inflammatory process leads to dysregulation of lipid metabolism and contributes to the worsening of metabolic syndrome variables. The study by Castellano-Castillo and colleagues [3] identified decreased DNA methylation of the gene encoding tumour necrosis factor (TNF) in subjects with metabolic syndrome compared to subjects without metabolic syndrome [3]. Moreover, negative correlations were found between TNF DNA methylation and metabolic syndrome index, body mass index, triglycerides, glucose, LDL-cholesterol and diastolic blood pressure, whereas a positive association was observed with HDL-cholesterol, indicating the roles of TNF in inflammation-associated deterioration of lipid metabolism [3].

TNF and many other inflammatory mediators were upregulated in insulin resistance and obesity [22]. Further, TNF receptor-deficient animals were protected against insulin resistance [22]. TNF was involved in impairment of insulin signalling by serine phosphorylation of IRS-1 and reducing GLUT4 expression [22]. TNF is expressed by adipose tissue and overexpressed in obesity, while acting as a modulator of insulin resistance in animal models [23]. TNF has also been linked to reduction in nitric oxide availability, potentially through reactive oxygen species generation and directly inhibiting endogenous nitric oxide synthase activity, leading to the development of endothelial dysfunction [23]. These mechanisms of TNF, beyond insulin resistance, play roles in the development of hepatocellular carcinoma and colorectal cancers [24].

Obesity has been associated with changes in gut microbiota, which is directly impacted by the dietary components [25,26]. Gut microbiota is capable of producing metabolites that have potential roles in epigenetic modifications, such as DNA methylation and histone modification. These metabolites include folate, riboflavin, niacin, pantothenic acid, pyridoxine and cobalamin [25,26,27]. These epigenetic mechanisms are highly relevant in early childhood, which is the phase for gut microbiota colonisation. Further, mode of delivery, breastfeeding, introduction of solid food, infections and antibiotic treatments play vital roles in the colonisation of gut microbiota [27]. Identifying the correlation between changes in gut microbiota and DNA methylation is an important research question that needs addressing.

Thus, the study of Castellano-Castillo and colleagues [3] has provided an excellent example of the importance of epigenetics in metabolic syndrome. This research shows that epigenetics may have a crucial role in both causing metabolic syndrome, as well as in therapeutic strategies to manage this world-wide health issue.
